# Metal-free carbon nanotubes: synthesis, and enhanced intrinsic microwave absorption properties

**DOI:** 10.1038/srep28310

**Published:** 2016-06-21

**Authors:** Xiaosi Qi, Jianle Xu, Qi Hu, Yu Deng, Ren Xie, Yang Jiang, Wei Zhong, Youwei Du

**Affiliations:** 1 Collaborative Innovation Center of Advanced Microstructures, Nanjing National Laboratory of Microstructures and Jiangsu Provincial Laboratory for Nano Technology, Nanjing University, Nanjing 210093, People’s Republic of China; 2 College of Science, Guizhou University, Guiyang 550025, People’s Republic of China

## Abstract

In order to clearly understand the intrinsic microwave absorption properties of carbon nanomaterials, we proposed an efficient strategy to synthesize high purity metal-free carbon nanotubes (CNTs) over water-soluble K_2_CO_3_ particles through chemical vapor decomposition and water-washing process. The comparison results indicated the leftover catalyst caused negative effects in intrinsic microwave absorption properties of CNTs, while an enhanced microwave absorption performance could be observed over the metal-free CNT sample. Moreover, the results indicated that the microwave absorption properties could be tuned by the CNT content. Therefore, we provided a simple route to investigate the intrinsic properties of CNTs and a possible enhanced microwave absorbing mechanism.

In order to prevent electromagnetic (EM) radiations caused by the rapid development of wireless communications and high frequency devices, microwave absorbing materials (MAMs) have attracted more and more attention all over the world^1^,^2^,^3^. It is well known that the reflection and attenuation properties of MAMs are mainly determined by the balance between the complex permittivity 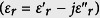 and the complex permeability 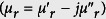. Because of the mismatch in the values of *μ*_*r*_ and *ε*_*r*_, it is very difficult to obtain a good matching on the single dielectric loss materials or magnetic loss materials^4^,^5^,^6^,^7^,^8^. Therefore, much research has been focused on core/shell structured nanohybrids (dielectric shells and magnetic nanoparticles as cores) as high efficiency MAMs, due to the synergetic effect between magnetic and dielectric losses^9^,^10^,^11^,^12^. Among these nanohybrids, core/shell structured magnetic nanoparticles and carbon-based including carbon nanomaterial (CNM) and graphene nanohybrids have received an increasing attention in recent years^13^,^14^,^15^,^16^,^17^,^18^. In order to explore high efficiency core/shell carbon-based nanohybrids, the EM parameters and microwave absorption properties of CNMs and graphene should be understood truly. Therefore, graphene and their derivatives were investigated intensively as potential microwave absorbers recently^19^,^20^. However, as we all know that CNMs such as carbon nanotubes (CNTs) and carbon nanofibers (CNFs) are usually synthesized by the methods of electric arc discharge, laser evaporation and catalytic chemical vapor deposition^21^,^22^,^23^. And the transition-metal catalysts are indispensable in these currently known methods, which makes the raw CNMs produced by these methods inevitably contain high concentration metal impurities. Moreover, because of the special physical and chemical properties of metal catalyst, the previous reported purification routes for CNMs are less effective and destructive^24^,^25^,^26^. Therefore, the intrinsic EM and microwave absorbing properties of CNMs still not be fully understood at present due to the leftover metal catalyst and the challenge of CNM purification^27^, which brings a huge obstacle to study and explore high efficiency carbon-based MAMs.

Therefore, the aim of this work is to synthesize high purity metal-free CNMs and investigate their intrinsic EM and microwave absorbing properties. Herein, based on the previous work^28^,^29^,^30^,^31^,^32^, we report a facile and efficient strategy to produce metal-free CNTs in large quantity over K_2_CO_3_ particles. Because the catalyst is water-soluble, the leftover catalyst particles can be removed completely from the raw CNMs through a very mild water washing process. Therefore, high purity and undamaged metal-free CNMs can be obtained by a simple and effective route, which can fulfill the investigation of the intrinsic properties of CNMs. Our results suggest that the microwave absorption performance of the CNT sample enhanced greatly after the removal of catalyst. And the possible CNT formation mechanism and enhanced microwave absorbing mechanism were discussed in details.

## Results

**Figure 1:**
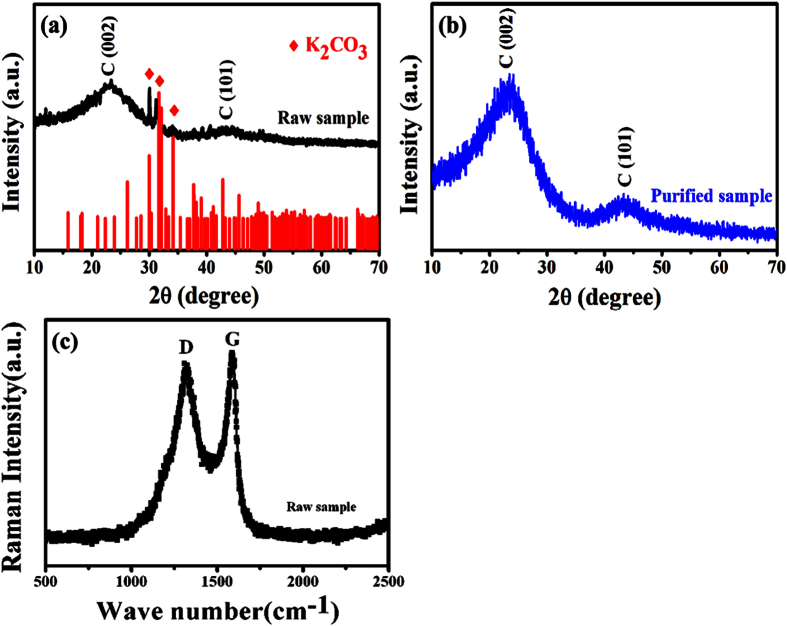
(**a,b**) XRD patterns, and (**c**) Raman spectra of the raw and purified samples.

Figure 1 gives the XRD patterns and Raman spectra of raw sample obtained over A-K_2_CO_3_ and the purified sample. As shown in Fig. 1a, all the diffraction peaks of raw sample are ascribable to graphite carbon and the corresponding catalyst K_2_CO_3_ (JCPDS: 71-1466). Because of its water-soluble property, the used catalyst should be removed easily and completely from the raw sample through the repeated washing processes. Figure 1b shows the XRD patterns of purified CNMs. One can find that all the peaks can be attributable to graphite carbon, and no signal assignable to the used catalyst can be detected over the purified sample. And the broad carbon peaks as shown in Fig. 1a,b indicate the relatively poor graphitic crystallinity of the obtained raw and purified samples. The comparison results imply that the catalyst particles can be removed effectively from the raw CNMs, and high purity CNMs can be obtained by this method. The graphitic property of the obtained sample can also be confirmed further by its Raman spectra. As shown in Fig. 1e, two peaks at ca. 1324 cm^−1^ (D band) and 1587 cm^−1^ (G band) can be observed clearly. It is well known that the D band can be attributed to the presence of sp^3^ defect within the carbon, and the G band is indicative of high crystallinity graphitic layer. Moreover, the intensity ratio of G and D bands (I_G_/I_D_) is usually used to characterize the crystallinity of CNMs. In our study, an I_G_/I_D_ of ca. 1.05 was recorded for the raw sample. Compared to CNMs reported previously^33^,^34^,^35^, the obtained sample exhibits a relatively low I_G_/I_D_ value, which displays its poor crystallinity. It is well known that the growth of CNMs is mainly determined by their experimental conditions such as the temperature, catalyst and so on. Therefore, compared to CNMs synthesized at high temperature or/and over the transition catalysts^33^,^34^,^35^, the relatively low I_G_/I_D_ value of the obtained CNMs should be related to the low pyrolysis temperature or/and inactive catalytic property of K_2_CO_3_.

**Figure 2:**
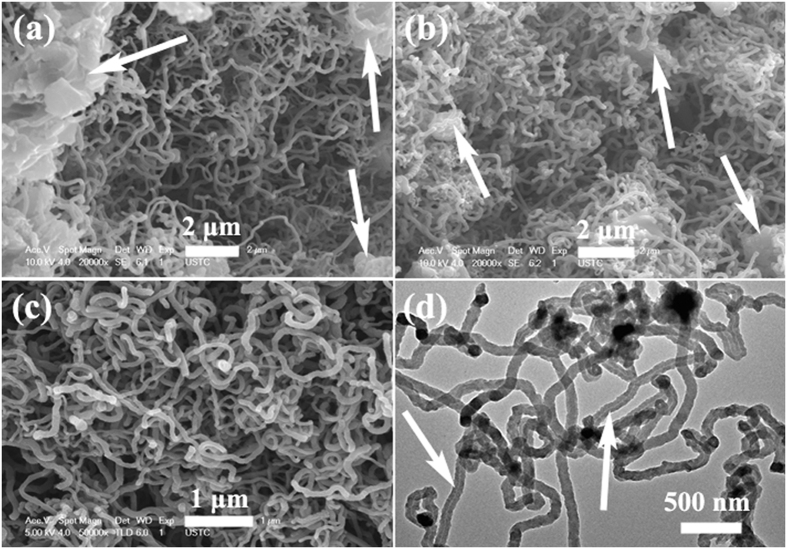
Microstructures of the obtained samples: (**a,b**) FE-SEM images of the raw sample, and (**c,d**) FE-SEM and TEM images of the purified sample.

In order to investigate the effect of purification process on the microstructures of raw CNMs, the FE-SEM and TEM images of raw sample obtained over A-K_2_CO_3_ and the purified samples are given in Fig. 2. As shown in Fig. 2a,b, CNTs are the majority in the obtained raw sample. The raw CNTs show a relatively uniform size (average diameter: ca. 80 nm). Moreover, besides high content of CNTs, different sizes of catalyst particles (as indicated by the arrows in Fig. 2a,b) can be observed clearly in the raw sample. Figure 2c shows the FE-SEM image of purified sample. After the repeated washing process, the catalyst particles cannot be seen and only CNTs can be observed in large scale. And the tube structure can be seen evidently by the closer TEM observation (as indicated by the arrows in Fig. 2d). Similar to the results reported before^31^,^36^, the comparison results indicate that the catalyst particles can be removed effectively from the raw sample, and the washing process does not bring any destruction on the morphology of the obtained CNTs.

**Figure 3:**
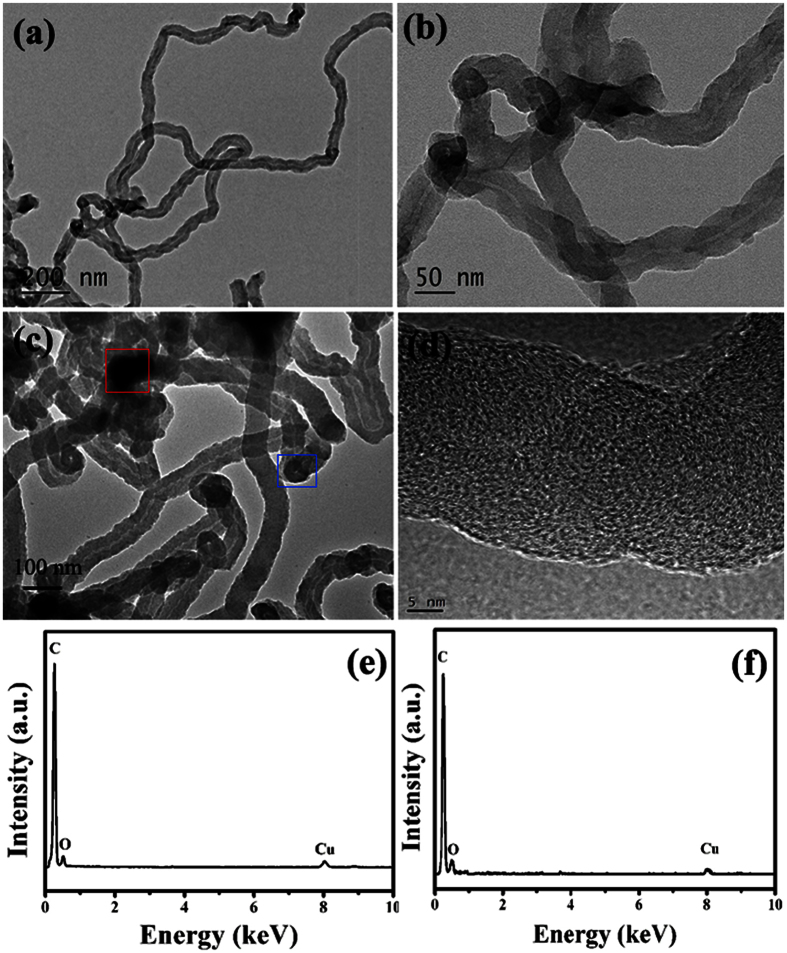
Electron microscopy characterization of the purified CNTs: (**a**) TEM image, (**b,c**) Enlarged TEM images, (**d**) HRTEM image, (**e,f**) EDS spectra from the area as indicated by the red and blue square in (**c**), respectively.

In order to confirm the obtained results of purified CNTs, detailed electron microscopy characterization of the purified CNTs was carried out and the results were shown in Fig. 3. As shown in Fig. 3a–c, one can observed clearly that the product displays the hollow tubular structure and the top section of CNTs appears as an onion-like structure. Moreover, no evident damages can be observed over the purified CNTs, and catalyst particle cannot be seen inside the obtained CNT. And the high resolution TEM (HRTEM) result (as shown in Fig. 3d) indicates the obtained CNTs exhibit the relatively low crystallinity of graphitic layer, which is consistent with the obtained XRD and Raman results. To prove the effective purification process, the energy dispersive X-ray spectroscopy (EDS) results of dark area and top end of CNTs are provided (as shown in Fig. 3e,f), respectively. The EDS results are obtained from the areas as indicated by the red and blue square in (c), respectively. One can find only C, O and Cu can be detected over the purified CNTs. In this study, we think that the C signal originates from CNT, Cu signal comes from the copper grid and the water-washing process induces the formation of O. Generally, because the used catalyst is water-soluble, the purification process here is simple, mild, low-cost, environment-friendly and effective, and the route may make the properties and applications of CNMs verified or realized fully.

**Figure 4:**
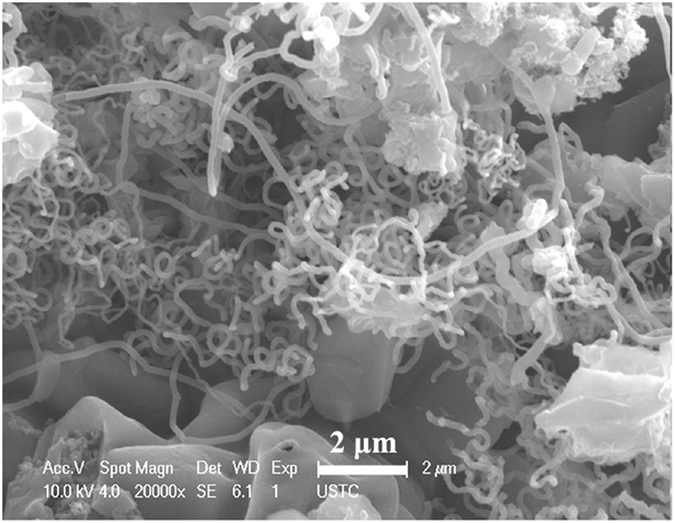
FE-SEM of the sample obtained over the B-K_2_CO_3_ particles.

In order to study the effect of catalyst preparation method on CNM growth, the obtained B-K_2_CO_3_ was used as catalyst for the decomposition of acetylene. With the other experimental conditions unchangeable, about 0.1 g of black sample could be collected in the ceramic plate. As shown in Fig. 4, one can find that the obtained sample at the case consists of CNTs with high selectivity and catalyst particles with different sizes. Compared to CNTs obtained over the catalyst A-K_2_CO_3_ particles, the leftover catalyst particles can be observed very frequently and big sizes of CNTs can be seen. To investigate the stability of the designed experiments, each experiment was repeated three times to confirm the obtained results. Generally, as shown in Table 1, one can find that the preparation method for catalyst has a great impact on the yield and size of the obtained CNTs, and the designed experiments show a good reproducibility. Moreover, the yield of CNTs obtained over A-K_2_CO_3_ is much higher than that of CNMs reported previously^31^,^37^,^38^.

According to the transmission line theory, the reflection loss (RL) and attenuation constant (***α***) were calculated by the following equation^39^,^40^,^41^:


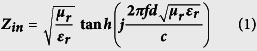



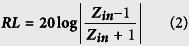



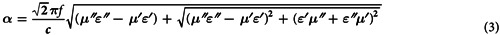


where ***f*** is the frequency of the EM wave, ***d*** is the thickness of an absorber, ***c*** is the velocity of light and ***Z***_***in***_ is the input impedance of absorber.

**Figure 5:**
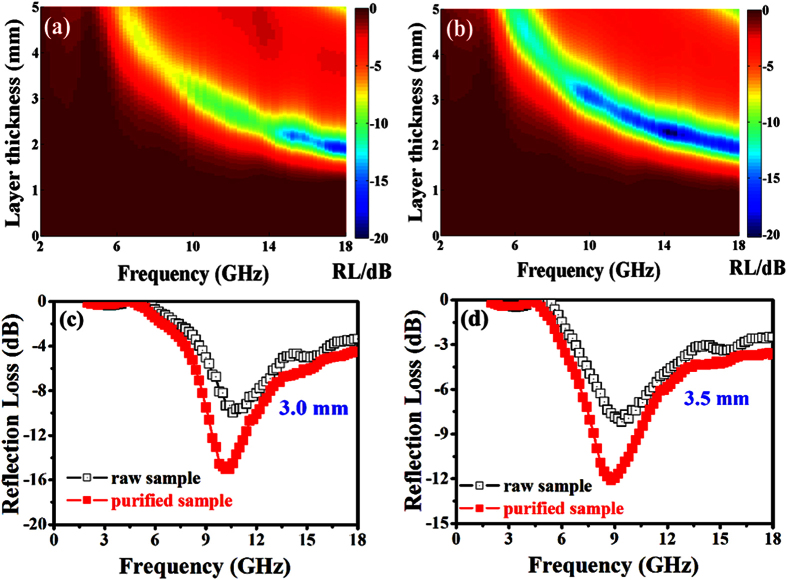
Microwave absorption characteristics of the raw and purified CNT (30 wt%) composites: (**a,b**) color map of the RL values, and (**c,d**) RL versus frequency with the thickness of 3.0 and 3.5 mm.

Based on Eqs (1) and (2), the RL values of the CNT composites containing 30 wt% of the raw or purified CNTs were calculated and the results are shown in Fig. 5. Figure 5a,b show the color map of RL values of raw and purified CNT samples. It is obvious that the minimum RL moves toward to the lower frequency region with an increasing thickness. A minimum RL value of −17.2, −20.2 dB was observed at 17.4, 14.2 GHz on the raw and purified CNT samples with a matching thickness of 1.93, 2.28 mm, respectively. RL values below −10 dB (90% of EM wave attenuation) can be obtained over the purified CNT sample in the frequency range of 5.8–18 GHz. Generally speaking, the purified CNT sample exhibits better microwave absorption ability than the raw one. Figures 5c,d shows a typical RL versus frequency for the raw and purified CNT samples with the thickness of 3.0 and 3.5 mm. One can find that the minimum RL is observed at the different frequencies with the same matching thickness. In general, the obtained result indicates that the remained catalyst particles cause a big gap between the measured and intrinsic properties of CNM. Moreover, the results show that the intrinsic microwave absorption ability of CNTs is superior to those of the previously reported graphene and their derivatives^42^,^43^.

**Figure 6:**
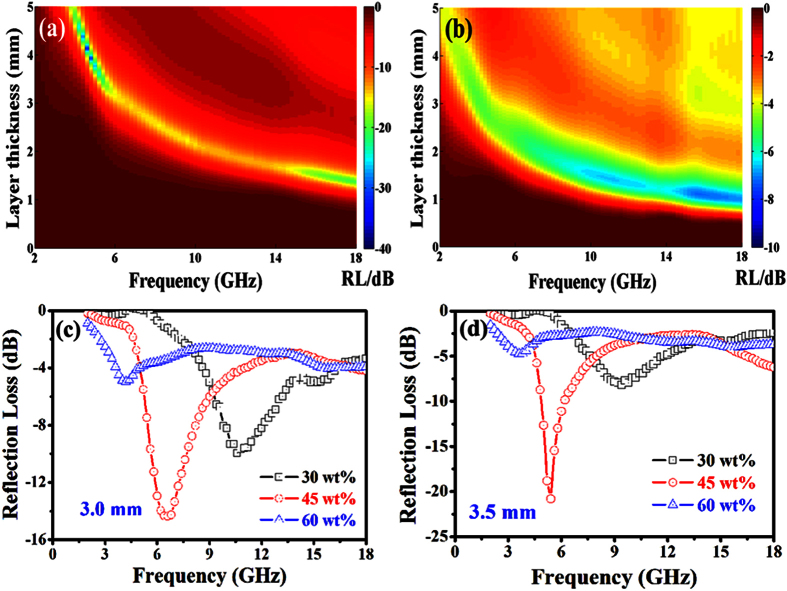
Microwave absorption characteristics of the raw CNT-paraffin composites with filler loading of (**a**) 45 wt%, (**b**) 60 wt%, and (**c,d**) RL versus frequency of the composites with the thickness of 3.0 and 3.5 mm.

Figure 6 shows the effect of CNT content on microwave absorption performance of the as-prepared CNT composites in the frequency range of 2 to 18 GHz. As shown in Fig. 6a, a minimum RL value of −32.7 dB is observed at 4.6 GHz for the CNT (45 wt%)-paraffin composite with a thickness of 4.13 mm. While a minimum RL value can reach −7.2 dB (as shown in Fig. 6b) at 15.8 GHz for the CNT (60 wt%)-paraffin composite with a thickness of 1.10 mm. As shown in Fig. 6c,d, the typical RL results for the CNT (30, 45 and 60 wt%)-paraffin composite with the thickness of 3.0 and 3.5 mm indicates further that the microwave absorption performance improves gradually with the increase of CNT content from 30 to 45 wt%. Nevertheless, degraded EM wave absorption ability is observed for the CNT (60 wt%)-paraffin composite. Moreover, the RL peak moves to the lower frequency region with the increasing CNT content in the as-prepared composites, which can be attributed to the enhancement of ***ε***_*r*_ as pointed out by Fan *et al.*^44^. Generally speaking, the results show that the microwave absorption abilities of the CNT composites can be tuned by the CNT content, and the similar results and possible reasons were reported previously^45^,^46^,^47^.

## Discussion

**Figure 7:**
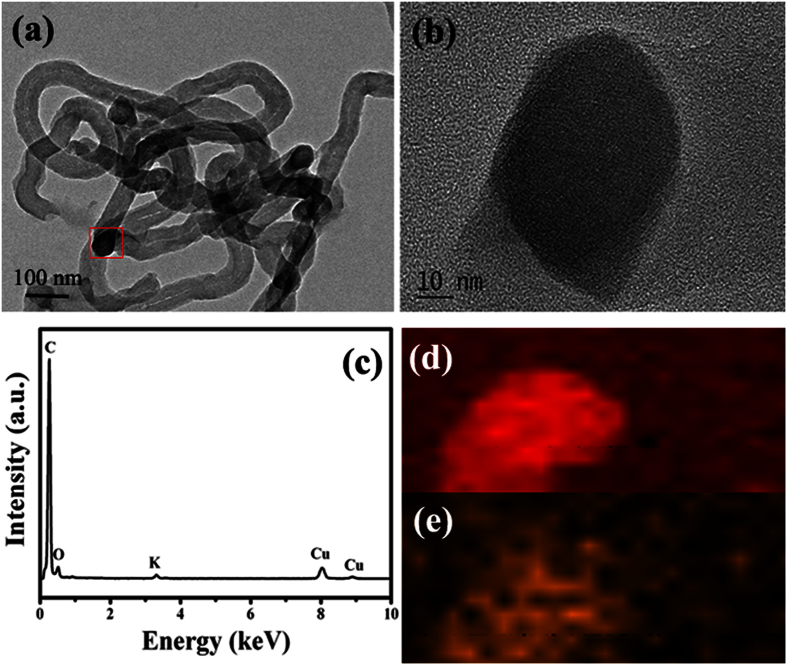
Microstructures of the raw sample: (**a**) TEM image; and (**b–e**) HRTEM image, EDS spectrum, EDS elemental mapping of C and K from the area as indicated by the red square in (**a**).

In order to understand the possible mechanism of K_2_CO_3_-catalyzed CNT growth, detailed TEM investigations were performed and the results were shown in Fig. 7. Figure 7 displays the microstructures of the raw CNTs without purification. Because of the water-soluble property, the obtained samples are very easy to deliquescence in the air. And the catalyst nanoparticles, which are encapsulated into the obtained CNTs, are very difficult to find. The same phenomenon was also reported before by Xu *et al.*^37^. Figure 7a,b presents the encapsulation of catalyst particle by CNT. And the results of EDS and element mapping (as shown in Fig. 7c–e) reveal that the top section of CNT is composed of C, O, Cu and K. As we all know that, the C signal originates from CNT and Cu signal comes from the copper grid. Therefore, the results give conclusive evidence that it is the K_2_CO_3_ nanoparticles that acts as the catalyst to catalyze the growth of CNTs. Moreover, based on the obtained TEM results (as shown in Fig. 3), one can find that the top section of purified CNTs appears as an onion-like structure should be related to the removal of catalyst particle after the water-washing process.

**Figure 8:**
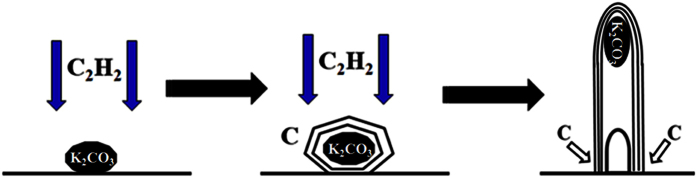
Schematic diagram for the possible formation mechanism of CNTs over K_2_CO_3_ nanoparticles.

It is well known that K_2_CO_3_ particles do not have the ability to decompose and react with carbon source such as acetylene. Based on the obtained results, the onion-like structures can be seen evidently at the top end of CNTs, which provides a direct evidence for the possible CNT growth mechanism. Same to the results reported by Xu *et al.*^37^, we also think the K_2_CO_3_ nanoparticles only act as a “seed” during the CNTs growth process. Based on the previously reported models and results^28^,^37^,^48^, the schematic illustration for the possible formation mechanism of CNTs over K_2_CO_3_ particles is given in Fig. 8. The possible pathways to grow CNTs are as follows: (1) the formation of carbon atoms through the decomposition of acetylene at relatively high temperature; (2) the K_2_CO_3_ nanoparticles provide the nucleation sites, and the generated carbon atoms nucleate on the surface of the K_2_CO_3_ nanoparticles; (3) the nucleated carbon atoms will assemble gradually over the K_2_CO_3_ nanoparticles; and (4) assemble of much more generated carbon atoms leads to the growth of CNTs.

**Figure 9:**
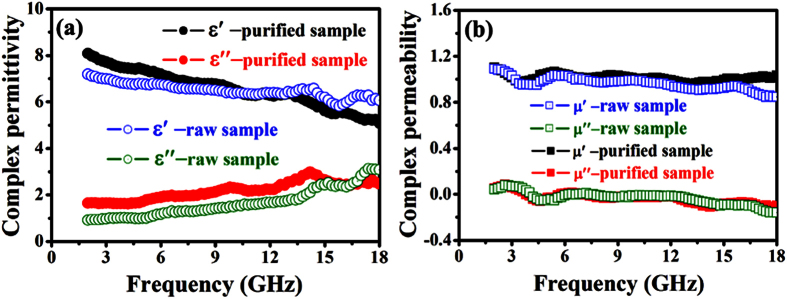
Frequency dependence of (**a**) complex permittivity, and (**b**) complex permeability for the raw and purified samples.

In order to analyze the difference in obtained RL results and probable absorption mechanism, the EM parameters, dielectric and magnetic loss ability, attenuation constant and impedance matching are presented. Figure 9 shows the variations of complex permittivity and permeability of the raw and purified CNT (30 wt%) samples with frequency. As illustrated in Fig. 9a, the values of the real (***ε*****′**) part of relative complex permittivity are found to decrease with the frequency in the tested frequency region. According to the Debye theory, ***ε*****′** can be described as:


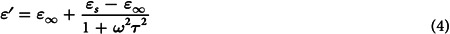


where ***ε***_***s***_ is the static permittivity, ***ε***_**∞**_ is the relative dielectric permittivity at the high frequency limit, ***ω*** is angular frequency, *τ* is polarization relaxation time. Based on the equation (4), one can find that the decrease of ***ε*****′** is mainly attributed to the increase of ***f***. As reported previously^47^,^49^, the phenomenon can be considered as the polarization relaxation in the lower frequency range. Obviously, the real (***ε*****′**) and imaginary (***ε*****″**) parts of relative complex permittivity of the purified CNT sample are slightly higher than those of the raw CNT sample. The increment of ***ε*****′** may be attributed to the fact that the removal of K_2_CO_3_ catalyst can increases the dipolar polarization^50^. According to ***ε″***** ∝ *****σ*****/2*****πε***_**0 **_***f***, the decreasing of the resistance (R) will lead to the increasing of dielectric loss. The CNT purification should enhance the conductivity (***σ***) of the sample due to the electrical conductivity of catalyst. Therefore, the difference in these values of complex permittivity should be related to the existence of the catalyst in the raw sample. Figure 9b shows the real (***μ*****′**) and imaginary (***μ*****″**) part of complex permeability obtained over the raw and purified CNT samples as a function of frequency. One can see that the former is close to 1.0 while the latter to 0. Because there are no magnetic particles in raw and purified CNT samples, the diversion of complex permeability can be negligible between the raw and purified CNT samples. Therefore, the leftover catalyst particles actually bring a great difficulty on the EM characterization of CNM.

**Figure 10:**
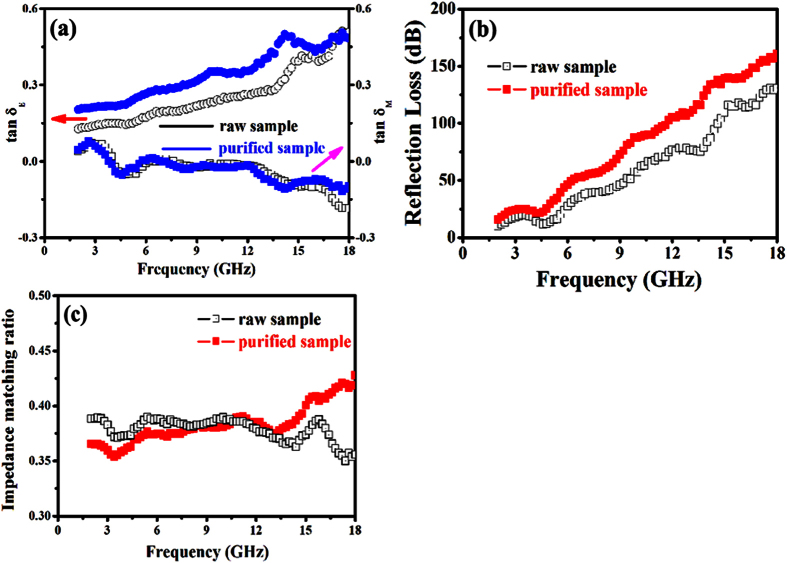
(**a**) Loss tangent, (**b**) attenuation loss, and (**c**) impedance matching of the raw and purified samples.

Based on the data of the measured complex permittivity and permeability (as shown in Fig. 9a,b), the dielectric tangent (**tan *****δ***_***E***_ = ***ε*****″/*****ε*****′**) and magnetic tangent loss (**tan *****δ***_***m***_ = ***μ*****″/*****μ*****′**) were calculated, and the result is shown in Fig. 10a. One can find that all the samples exhibit much higher **tan*****δ***_***E***_ values than those of **tan *****δ***_***m***_ in the whole frequency range, which indicating that the dielectric loss plays the main role in the EM absorption. Moreover, it can be found clearly that the dielectric loss ability of the CNTs enhanced greatly after the purification of catalyst, and the magnetic tangent loss ability is almost unchangeable due to the nonmagnetic property of catalyst. As the papers reported recently^51^,^52^,^53^, the enhanced microwave absorption properties mainly resulted from the attenuation constant (***α***) and impedance matching. According to equation (3), the obtained ***α*** values of the raw and purified CNTs in the entire frequency range are shown in Fig. 10b. The attenuation loss ability of the purified CNTs is evidently superior to the raw CNTs, which showing that the microwave absorption properties may be enhanced through the catalyst purification process. The impedance matching ratios of the obtained samples were provided in Fig. 10c. It is clearly seen that the purified CNTs exhibits much better impedance matching properties at the higher frequency range (ca. 10.0–18.0 GHz). Generally, the enhanced microwave absorption abilities of the purified CNTs can be ascribed to the tradeoff among the dielectric and magnetic loss ability, attenuation constant and impedance matching.

**Figure 11:**
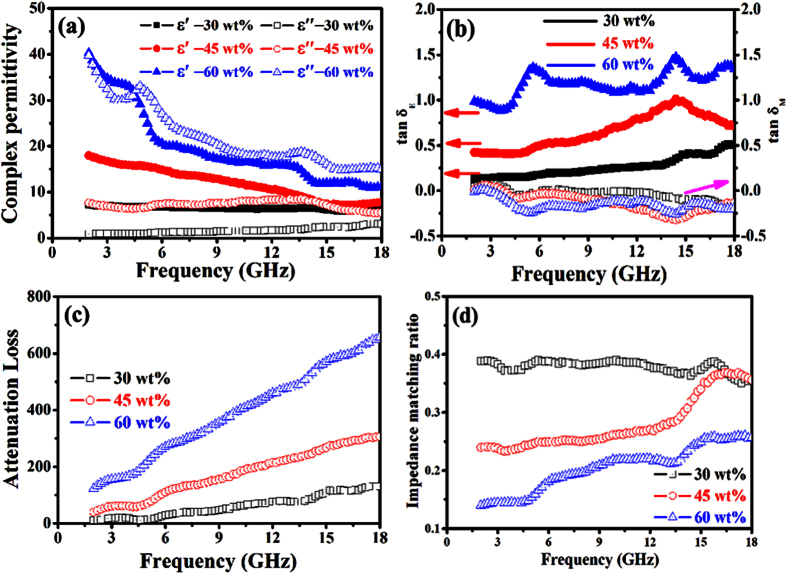
(**a**) Complex permittivity, (**b**) loss tangent, (**c**) attenuation loss, and (**d**) impedance matching of the CNTs-paraffin composites with filler loading ranging from 30 to 60 wt%.

In order to understand the difference in obtained RL results, the EM properties, dielectric and magnetic loss ability, attenuation constant and impedance matching of the as-prepared composites with the different filler contents were obtained. Figure 11a shows the complex permittivity of the as-prepared composites. These composites present a typical frequency dependent permittivity, the values of ***ε′*** decrease with the frequency in the whole frequency range. And significant enhancement is achieved in both ***ε′*** and ***ε″*** with the increase of CNT loading ranging from 30 to 60 wt%, which is similar to the other composites reported before^45^,^46^,^47^. The enhancement of ***ε***_***r***_ confirms further the shift of obtained RL peak (as shown in Fig. 6c,d) with the increasing CNT content in the as-prepared composites. Figure 11b presents the dielectric tangent and magnetic tangent loss of the as-prepared composites. The CNT composites exhibit enhanced **tan*****δ***_***E***_ values with the increasing CNT content. And the **tan*****δ***_***M***_ values are almost unchangeable when the CNT content increases. Figure 11c displays the calculated ***α*** values of the as-prepared composites. It can be seen clearly that the value of ***α*** increases with the CNT content. The impedance matching ratio of the as-prepared composites is presented in Fig. 11d. With the increasing CNT content from 30 to 60 wt%, the impedance matching ability of the CNT composites is getting worse. It is well known that the enhanced microwave absorption performance mainly can be ascribed to the good impedance matching ratio, high values of ***α***, **tan*****δ***_***E***_ and **tan*****δ***_***M***_, good compensation between the dielectric loss and magnetic tangent loss. Based on the aforementioned results, one can found that the enhanced microwave absorption abilities of the CNT (45 wt%) composite can also be attributed to the tradeoff among the dielectric and magnetic loss ability, attenuation constant and impedance matching, which is similar to the recently reported Co_x_Fe_y_@C composites^51^.

In summary, we propose an efficient strategy to synthesize metal-free CNTs through the chemical vapor deposition and water washing process. The studies on the microstructures of the obtained samples indicate that the K_2_CO_3_ nanoparticles serve as seeds and provide the nucleation sites for CNT growth. The investigation of microwave absorption properties indicates that the leftover catalyst causes problems in intrinsic property characterization of CNTs, and an enhanced microwave absorption performance can be found over the purified sample. Moreover, the obtained results indicate that the microwave absorption properties of the as-prepared composites can be tuned by the CNT content. The enhanced microwave absorption performance of the CNT composite can also be attributed to the tradeoff among the dielectric and magnetic loss ability, attenuation constant and impedance matching. Therefore, we propose a simple and effective route to study the intrinsic properties of CNMs and their possible enhanced microwave absorption mechanism.

## Methods

### Catalyst preparation

All the materials used here were commercially available and analytically pure. In order to study the effect of catalyst preparation method on the growth of CNMs, the catalyst K_2_CO_3_ particles could be generated by the two different methods. In the first typical method, 0.1 mol KOH and 0.1 mol oxalic acid were dissolved in 200 ml of absolute alcohol. After stirring at 60 °C for 6 h, the mixture was kept at 80 °C for several hours until the formation of a white powder. The obtained powder was heated twice in air at 550 °C for 4 h and the catalyst was obtained. For distinguish, the K_2_CO_3_ generated by this method is denoted as A-K_2_CO_3_. And the catalyst K_2_CO_3_ particles could also be obtained as follows: (i) firstly, the purchased commercial and analytically pure K_2_CO_3_ was dissolved in deionized water; (ii) then the solution was kept at 80 °C for several hours until the formation of a white powder. The K_2_CO_3_ produced by this method is denoted as B-K_2_CO_3_.

### Generation of CNTs

In the typical experiment, 0.1 g of the obtained white powder (A-K_2_CO_3_ or B-K_2_CO_3_) was dispersed on a ceramic plate that was placed inside a quartz tube. After that, the temperature of the furnace was raised from room temperature (RT) to 450 °C with Ar flowing through the reaction tube. Then shutting off Ar, acetylene was introduced into the tube at 450 °C for 6 h at atmospheric pressure. After cooling to RT, about 1.1 g of black sample can be obtained in the ceramic plate. In order to obtain high purity carbon nanomaterials (CNMs), the obtained raw black sample was purified through the repeated washing process.

### Measurement

The samples were examined on an X-ray powder diffractometer (XRD) at RT for phase identification using CuK_α_ radiation (model D/Max-RA, Rigaku). Raman spectroscopic investigations were performed using a Jobin-Yvon Labram HR800 instrument with 514.5 nm Ar^+^ laser excitation. The morphologies of the samples were examined using a transmission electron microscope (model JEM-2000EX, operated at an accelerating voltage of 200 kV), and a field emission scanning electron microscope (FE-SEM) (model FEI Sirion 200, operated at accelerating voltages of 5 kV). For microwave measurement, the as-prepared CNTs obtained over the catalyst A-K_2_CO_3_ and purified CNTs were mixed with paraffin. The relative complex permittivity (***ε***_***r***_** = *****ε′*** − ***jε*****″)** and permeability (***μ***_***r***_** = *****μ′*** − ***jμ*****″)** of the composite were measured in frequency range of 0.5–18 GHz over an Agilent E8363B vector network analyzer.

## Additional Information

**How to cite this article**: Qi, X. *et al.* Metal-free carbon nanotubes: synthesis, and enhanced intrinsic microwave absorption properties. *Sci. Rep.* **6**, 28310; doi: 10.1038/srep28310 (2016).

## Figures and Tables

**Table 1:** Effect of the catalyst preparation method on the CNM growth.

Catalyst	A-K_2_CO_3_	B-K_2_CO_3_
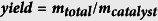	11.02	1.01
	10.98	1.04
	11.03	1.02
**TEM studies**	CNTs	CNTs
**Size of carbon species**	60–100 nm	60–200 nm
